# Tigecycline as salvage treatment of febrile neutropenia in patients with haematological malignancies—a retrospective single-centre analysis of 200 cases

**DOI:** 10.1007/s00277-023-05222-5

**Published:** 2023-04-25

**Authors:** Daniel Geßner, Mirjeta Berisha, Torben Esser, Enrico Schalk

**Affiliations:** 1 Department of Haematology and Oncology, Otto-von-Guericke University Magdeburg, Medical Centre, Leipziger Str. 44, 39120 Magdeburg, Germany; 2 Institute of Medical Microbiology and Hospital Hygiene, Otto-von-Guericke University Magdeburg, Medical Centre, Magdeburg, Germany

**Keywords:** Tigecycline, Febrile neutropenia, Haematological malignancy, Chemotherapy, Antibiotics

## Abstract

Tigecycline has been used to treat patients with febrile neutropenia (FN). This study aims to analyse the effectiveness of tigecycline as salvage treatment of FN. Patients records from 09/2004 to 04/2019 were reviewed. Cases were eligible if fever persisted/recurred (p/r-FN) after 3 days of second-line treatment with a carbapenem, and were divided into three groups: switch to tigecycline (TGC group), switch to other antibiotics (OAB group), and no switch (W&W group). The primary endpoint was response rate (defervescence for ≥ 7 days or at least until discharge); the key secondary endpoint was 30-day mortality rate. Two hundred cases from 176 patients (median 59 years; 53.5% men) treated were included, mostly acute myeloid leukaemias (61.0%). 45.5% of cases were in the TGC group (in combination with an anti-pseudomonal antibiotic, mostly ceftazidime [95.6%]); 35.5% were in the OAB and 19.0% in the W&W group. There was no significant difference in response rates (TGC, 73.6%; OAB, 62.0%; W&W, 78.9%; *p* = 0.12) or 30-day mortality rates (TGC, 7.7%; OAB, 7.0%; W&W, 5.3%; *p* = 0.94). Tigecycline plus an anti-pseudomonal antibiotic does not improve response or 30-day mortality rate compared to other antibiotics in patients with p/r-FN. Also, in some cases, no switch in antibiotics may be necessary at all.

**Supplementary Information:**

The online version contains supplementary material available at 10.1007/s00277-023-05222-5.

## Introduction

Neutropenia is a common side effect after chemotherapy. In particular, severe neutropenia with an absolute neutrophil count (ANC) of < 0.5 × 10^3^/µL increases the risk of infectious complications. The risk of febrile neutropenia is high in curative treatment of aggressive lymphoma (20–40%), acute leukaemia (≥ 40%), and following autologous or allogeneic haematopoietic stem cell transplantation (HSCT) (up to 100%) [1, 2]. Anti-pseudomonal broad-spectrum ß-lactam antibiotics are used for initial treatment in high-risk patients. Piperacillin/tazobactam, meropenem, and imipenem have comparably good response rates, while ceftazidime and cefepime were shown to be inferior [3, 4]. The median time to fever resolution is 4–5 days with initial therapy [5]. However, some patients develop fever or other signs of infection again during the initial treatment. The pathogen spectrum may be altered and antibiotic resistance is probably increased [6]. If fever persists after 72–96 h of antibiotic therapy, antifungal treatment should be added [7]. For clinically unstable patients, a change in antibiotic therapy should be considered, e.g. escalation to carbapenems, if not done initially, or adding aminoglycosides or glycopeptides to therapy [8]. For clinically stable patients, it is recommended to continue first-line antibiotic therapy unchanged.

Tigecycline, a tetracycline derivative, is the first approved drug from the glycylcycline antibiotic group and has a broad spectrum of activity in the Gram-positive and Gram-negative spectrum, including against *Staphylococcus aureus*, coagulase-negative staphylococci (CoNS), *Escherichia coli*, *Klebsiella pneumoniae* and *Enterococcus* spp., as well as against methicillin-resistant *S. aureus* and vancomycin-resistant enterococci. It is mainly bacteriostatic with partial bactericidal activity against *Streptococcus pneumoniae*. The main exception is *Pseudomonas aeruginosa*, therefore tigecycline should always be combined with an anti-pseudomonal antibiotic in neutropenic patients [9].

Tigecycline was approved for the treatment of complicated intra-abdominal, as well as skin and soft tissue infections. However, tigecycline has also been shown to be effective in treating bloodstream infections and pneumonia in immunocompetent patients [10, 11]. Some studies have also demonstrated efficacy for cancer patients. In two retrospective and one prospective analysis, tigecycline was effective in the treatment of febrile neutropenia in second- and third-line therapy [12–14].

Febrile neutropenia can be potentially life-threatening, and the use of reserve antibiotics for multidrug-resistant fever is not unusual in patients with haematological malignancies. After two lines of a multidrug treatment, most microorganisms should be covered; nevertheless, some patients do not respond and show no resolution of fever. This cohort with multidrug-resistant febrile neutropenia should be investigated in our study. Why neutropenic patients have ongoing fever after first- or second-line therapy can have several reasons [15], e.g. drug-resistance, fungal or viral infections, other infections or non- infectious diseases, such as drug fever.

The aim of this study was to analyse the effectiveness of tigecycline in patients with multidrug-resistant persistent or recurrent (p/r) fever after chemotherapy-induced neutropenia after failure of second-line treatment with a carbapenem.

## Patients and methods

**Fig. 1:**
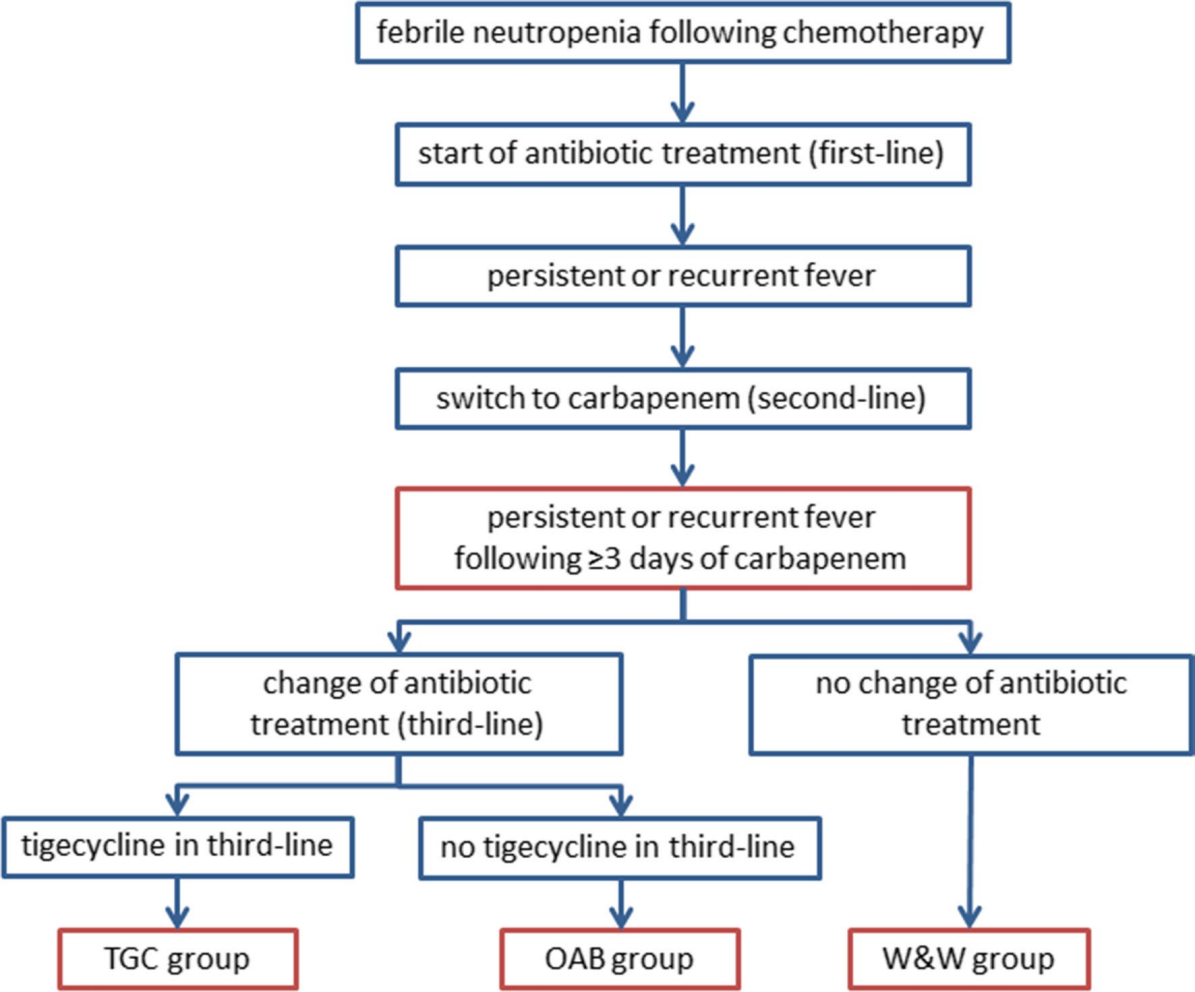
Treatment algorithm. *OAB* other-antibiotics, *TGC* tigecycline, *W&W* watch & wait

In this retrospective study, we investigated the internal database for adult patients with p/r fever after chemotherapy-induced neutropenia, treated at the Department of Haematology and Oncology, University Hospital Magdeburg, Magdeburg, Germany. p/r fever was defined as a body temperature of ≥ 38.3 °C or ≥ 38.0 °C lasting for at least 1 h following a minimum of 3 days of second-line antibiotic treatment with a carbapenem. Temperature was usually measured axillary. Patients were divided into three groups: tigecycline (TGC) group, if tigecycline was given as part of third-line therapy; other-antibiotics (OAB) group, if patients received any other antibiotics as third-line therapy or were added to the current treatment with carbapenems, but not tigecycline; or watch & wait (W&W) group, if no third-line therapy was given and second-line treatment was continued without change. The treatment algorithm is shown in Fig. 1. Tigecycline was administered in the approved standard dose of 100 mg as loading dose, followed by 50 mg every 12 h. Regarding antifungal treatment, no clear strategy was followed: Antifungals were administered either empiric or pre-emptive. In the time before the approval of posaconazole prophylaxis regularly aspergillus galactomannan screening in high-risk patients was performed, added by computer tomography scan of the lung in cases of antibiotic refractory fever or respiratory symptoms; antifungal therapy was administered in cases of positive aspergillus antigen tests or detection of lung infiltrates. After introduction of posacoanazole prophylaxis, no aspergillus antigen screening was performed. Antifungal therapies were administered in cases of antibiotic refractory fever or detection of lung infiltrates.

Neutropenia was defined as an ANC < 0.5 × 10^3^/µL or leucocytes < 1.0 × 10^3^/µL when no ANC was available. The cause of p/r fever was classified as microbiologically diagnosed infections (MDI), lung infiltrates (LI) or fever of unknown origin (FUO). MDI was defined as a positive microbial culture in blood or bronchoalveolar fluid. In the case of CoNS or other known skin contaminants two positive blood cultures were required. Central venous catheter-related bloodstream infections (CRBSI) were diagnosed according to the 2012 Infectious Diseases Working Party (AGIHO) of the German Society of Haematology and Medical Oncology (DGHO) definitions [16]. Positive results from polymerase chain reaction (PCR) for viral or aspergillus galactomannan antigen for invasive fungal infections (IFI) were also counted as MDI. Radiologically confirmed LI were defined as cause of fever regardless of symptoms. IFI were accessed using the European Organization for Research and Treatment of Cancer/Invasive Fungal Infections Cooperative Group and the National Institute of Allergy and Infectious Diseases Mycoses Study Group (EORTC/MSG) Consensus Group 2008 definitions [17]. If MDI and LI were diagnosed simultaneously, the case was classified as MDI. All cases not diagnosed as MDI or LI were evaluated as FUO.

The primary endpoint was response rate, defined as defervescence < 38 °C for ≥ 7 days or at least until discharge. Time to begin of defervescence had to be ≤ 5 days after initiation of third-line therapy (TGC and OAB group) or failure of second-line treatment (W&W group). The key secondary endpoint was 30-day mortality rate.

### Statistical analysis

For differences, Pearson's χ^2^ test or Kruskal–Wallis test were used where appropriate. A two-sided *p* value of < 0.05 was deemed significant. Statistical analysis was done by using SPSS version 25 (IBM®, Armonk, NY, USA).

## Results

### Patient characteristics

A total of 200 episodes with p/r fever from 176 patients treated from 09/2004 to 04/2019 were included (Supplementary Figure 1). Median age was 59 years and 53.5% of patients were male. Eastern Cooperative Oncology Group (ECOG) performance score (PS) at start of inpatient treatment was 0–1 in 173 cases (96.1%). In 20 cases, no ECOG PS was documented. All patients had received myelosuppressive or myeloablative chemotherapy before the febrile episode. All patients were neutropenic at some point following chemotherapy and in 168 cases (84.0%), the patients were still neutropenic at the beginning of p/r fever. The median duration of neutropenia was 21 days (range 1–67 days). The most common diagnosis was acute myeloid leukaemia (AML) with 122 cases (61.0%). Of the 200 cases, 91 cases (45.5%) were included in the TGC group, 71 cases (35.5%) in the OAB group, and 38 cases (19.0%) in the W&W group. Most patients (70.0%) were treated with myelosuppressive chemotherapy without any stem cell support. Autologous HSCT was performed in 32 cases (16.0%) and allogeneic HSCT was performed in 28 cases (14.0%). There were significantly more cases of allogeneic HSCT in the TGC group than in the OAB group and W&W group (TGC, 25; OAB, 2; W&W, 1; *p* < 0.001). As shown in Table 1, there was no significant difference in age, sex, haematological/oncological diagnosis, remission status or rate of neutropenia between the three treatment groups.

**Table 1:** Patient characteristics

Characteristic	TGC	OAB	W&W	*p* value
Cases, *n*	91	71	38	
Patients, *n*	82	69	35	
Age, years				0.88^a^
Median	59	58	59.5	
Range	18–74	19–77	18–75	
Male, *n* (%)	50 (54.9)	34 (47.9)	23 (60.5)	0.42^b^
ECOG PS, *n* (%)^c^				0.94^b^
0–1	87 (95.6)	58 (96.7)	28 (96.6)	
2–4	4 (4.4)	2 (3.3)	1 (3.4)	
Diagnosis, *n* (%)				0.27^b^
AML	52 (57.1)	48 (67.6)	22 (57.9)	
ALL	16 (17.6)	5 (7.0)	5 (13.2)	
NHL	11 (12.1)	14 (19.7)	6 (15.8)	
MM	7 (7.7)	2 (2.8)	2 (5.3)	
HL	1 (1.1)	1 (1.4)	2 (5.3)	
MDS/MPN	4 (4.4)	0	0	
GCT	0	1 (1.4)	1 (1.4)	
Remission status, *n* (%)				0.36^b^
Initial diagnosis	26 (28.6)	35 (49.3)	17 (44.7)	
Complete remission	33 (36.3)	21 (29.6)	12 (31.6)	
Partial remission	13 (14.3)	6 (8.5)	3 (7.9)	
Relapse	10 (10.9)	4 (5.6)	3 (7.9)	
Refractory disease	9 (9.9)	5 (7.0)	3 (7.9)	
Chemotherapy, *n* (%)				< 0.001^b^
Conventional CTx	54 (59.3)	57 (80.3)	29 (76.3)	
Autologous HSCT	12 (13.2)	12 (16.9)	8 (21.1)	
Allogeneic HSCT	25 (27.5)	2 (2.8)	1 (2.6)	
Neutropenia, *n* (%)^d^	72 (79.1)	62 (87.3)	34 (89.5)	0.22^b^
Duration of neutropenia, days				0.10^a^
Median	22	19	17	
Range	1–67	2–57	4–37	
Cause of p/r fever, *n* (%)				0.07^b^
MDI	14 (15.4)	14 (19.7)	14 (36.8)	
LI	38 (41.8)	24 (33.8)	13 (34.2)	
FUO	39 (42.9)	33 (46.5)	11 (28.9)	

*ALL* acute lymphatic leukaemia, *AML* acute myeloid leukaemia, *CTx* chemotherapy, *ECOG PS* Eastern Cooperative Oncology Group performance score, *FUO* fever of unknown origin, *GCT* germ cell tumour, *HL* Hodgkin lymphoma, *HSCT* haematopoietic stem cell transplantation, *LI* lung infiltrates, *MDI* microbiologically documented infection, *MDS* myelodysplastic syndrome, *MM* multiple myeloma, *MPN* myeloproliferative neoplasm, *NHL* non-Hodgkin lymphoma, *OAB* other-antibiotics, *p/r* persistent or recurrent, *TGC* tigecycline, *W&W* watch & wait

^a^Kruskal-Wallis test

^b^Pearson's χ^2^ test

^c^in 11 cases (OAB group) and 9 cases (W&W group), no data was available regarding ECOG PS

^d^after failure of second-line treatment

### Cause of p/r fever

MDI was diagnosed in 42 cases (21.0%) with 14 cases in each treatment group. In 38 of these cases, the diagnosis was determined by a positive microbial blood culture. Of the 42 cases with MDI, 22 (52.4%) also had radiologically documented LI but were counted as MDI cases. In 5 cases, two species were identified in one blood culture. Gram-positive bacteria were found in 33 cases (86.8%) und Gram-negative in 10 cases (26.3%). The most common bacteria were CoNS (15 cases), *Enterococcus* spp. (12 cases), *E. coli* (4 cases) and *P. aeruginosa* (4 cases)*.* The isolated bacteria from blood cultures are shown in Table 2. In 6 of 43 positive blood culture cases (14.0%) *definite* CRBSI were diagnosed. The remaining MDI were 1 case of *Stenotrophomonas maltophilia* from bronchoalveolar lavage (BAL), 1 case of *Pneumocystis jirovecii* from BAL and 2 cases of influenza A (1 from nasopharyngeal swab and 1 from BAL). Four isolates of bacteria were multidrug resistant: *E. coli* (2 cases), *P. aeruginosa* (1 case) and *S. maltophilia* (1 case). In the TGC group, 7 of the 14 species were sensitive to tigecycline while 2 more are suspected to be sensitive. Four cases of *P. aeruginosa* were naturally resistant to tigecycline. Fourteen of the 16 isolates in the OAB group and 11 of the 14 isolates in the W&W group were tested to be sensitive to a least one of the antibiotics given. More details are given in the Supplementary Table 1. LI were diagnosed in 75 cases (37.5%), with 38 cases (41.8%) in the TGC group, 24 cases (33.8%) in the OAB group and 13 cases (34.2%) in the W&W group. The remaining 83 cases (41.5%) were evaluated as FUO. There was no significant difference in the cause of p/r fever between the 3 treatment groups (*p* = 0.07). Among the 200 cases studied, we found *proven*, *probable* and *possible* IFI in 1.0% (2/200; *Mucorales* in all), 2.0% (4/200; *Aspergillus* spp. in all) and 13.0% (26/200) of the cases, respectively. Of these patients, only one patient with possible IFI from the OAB group died. He received voriconazole and cause of death was sepsis.

**Table 2:** Results of blood cultures

Bacteria	TGC *n* = 13	OAB *n* = 16	W&W *n* = 14
Gram-positive, n	9	16	8
*Bacillus cereus*	0	1	0
*Enterococcus faecalis*	0	1	0
*Enterococcus faecium*	4	5	1
*Enterococcus gallinarum*	0	1	0
*Staphylococcus aureus*	1	0	2
*Staphylococcus epidermidis*	1	5	2
*Staphylococcus haemolyticus*	3	3	1
*Streptococcus dysgalactiae*	0	0	1
*Streptococcus mitis*	0	0	1
Gram-negative, n	4	0	6
*Escherichia coli*	0	0	4
*Klebsiella oxytoca*	0	0	1
*Proteus mirabilis*	0	0	1
*Pseudomonas aeruginosa*	4	0	0

*OAB* other-antibiotics, *TGC* tigecycline, *W&W* watch & wait

### Antibiotic and antifungal treatment

In second-line therapy, 85 cases (42.5%) were treated using monotherapy. In the OAB group, significantly more patients received monotherapy (TGC, 33.0%; OAB, 59.2%; W&W, 34.2%; *p* = 0.002). All patients were treated with a carbapenem as second-line therapy due to the study design. Meropenem was used in 198 cases (99.0%). The remaining 2 patients received imipenem. The most common antibiotics combined with a carbapenem were vancomycin and aminoglycosides. Numerically, vancomycin was used more often in the TGC group (TGC, 50.5%; OAB, 25.3%; W&W, 36.8%) but altogether the antibiotics used in second-line therapy were not significantly different between the three treatment groups (*p* = 0.11). Median duration of second-line therapy was 8 days in the TGC group, 6 days in the OAB group and 12 days in the W&W group. The median time from initiation of second-line therapy to begin of p/r fever was 4 days in the TGC and OAB group, and 3 days in the W&W group. The antibiotics used according to treatment group are listed in Table 3.

**Table 3:** Antibiotic treatment

Antibiotic	TGC *n* = 91	OAB *n* = 71	W&W *n* = 38	*p*-value
Second-line therapy, *n* (%)				0.11^a^
Meropenem	89 (97.8)	71 (100)	38 (100)	
Vancomycin	46 (50.5)	18 (25.3)	14 (36.8)	
Aminoglycoside	12 (13.2)	3 (4.2)	6 (15.8)	
Quinolone	5 (5.5)	4 (5.6)	3 (7.9)	
Macrolide	7 (7.7)	1 (1.4)	2 (5.2)	
Other	4 (4.4)	6 (8.5)	6 (15.8)	
Third-line therapy, *n* (%)				
Tigecycline	91 (100)	0		)^b^
Ceftazidime	87 (95.6)	15 (21.1)		
Meropenem	2 (2.2)	39 (54.9)		
Piperacillin/tazobactam	0	15 (21.1)		
Vancomycin	5 (5.5)	33 (46.5)		
Aminoglycoside	9 (9.9)	11 (15.5)		
Quinolone	11 (12.1)	10 (14.1)		
Macrolide	7 (7.7)	11 (15.5)		
Other	4 (4.4)	12 (16.9)		

*OAB* other-antibiotics, *TGC* tigecycline, *W&W* watch & wait

^a^Pearson's χ^2^ test

^b^no *p* value given because the difference in antibiotic treatment was determined by the study design

Of the 200 cases, 162 (81.0%) received third-line therapy (TGC and OAB group) while in the remaining 38 cases (19.0%), the second-line treatment was continued (W&W group). Only 6 cases (3.7%) were treated with monotherapy in the OAB group. Ceftazidime was the most commonly used ß-lactam antibiotic in third-line therapy and was used more often in the TGC -group (95.6%) than in the OAB group (21.1%). In the OAB group, more cases were treated with meropenem (54.9 vs. 2.2%) and piperacillin/tazobactam (21.1 vs. 0%) than in the TGC group. Vancomycin was used more often in the OAB group (46.5 vs. 5.5%). Median time from begin of p/r fever to initiation of third-line therapy was 0 days in the TGC and OAB -group. Median duration of third-line therapy was 9 days (range 3–25) in the TGC group and 8 days (range 4–24) in the OAB group.

In 178 cases (89.0%), antifungal treatment was given. There was no difference between the three treatment groups (TGC, 89.0%; OAB, 88.7%; W&W, 89.5%). The most commonly used antimycotics were voriconazole, liposomal amphotericin B and caspofungin.

### Response and 30-day mortality

The overall response rate was 70.5%. As shown in Table 4, there was no significant difference in the response rates between the three treatment groups (TGC, 73.6%; OAB, 62.0%; W&W, 78.9%; *p* = 0.12). In patients, who responded to therapy, median time to defervescence was 2 days in the TGC group (range 1–5), as well as in the OAB group (range 0–5) and 1 day (range 1–5) in the W&W group but the difference was not significant (*p* = 0.58). A total of 14 patients (7.0%) died. The 30-day mortality rate was 7.7% in the TGC group (7 patients), 7.0% in the OAB group (7 patients) and 5.3% in the W&W group (2 patients). The difference was not statistically significant (*p* = 0.94). Cause of death was infection-related in 11 cases and 3 patients died due to progressive haematological/oncological disease. Patients, unresponsive to antibiotic treatment, had a higher 30-day mortality rate than those responding (13.6 vs. 4.3%; *p* = 0.03). Neutropenic patients tended to have a lower response rate than non-neutropenic patients but the difference was not significant (67.9 vs. 84.4%; *p* = 0.09). Neutropenia had no significant effect on 30-day mortality. Interestingly, in patients ≥ 60 years response rates were higher than in younger patients (80.6 vs. 61.7%; *p* = 0.005) but 30-day mortality was not significantly different. In cases of FUO, response rates were higher than in cases with MDI or LI (MDI, 59.5%; LI, 58.7%; FUO, 86.7%; *p* < 0.001). This difference was also significant in the TGC group (MDI, 57.1%; LI, 63.2%; FUO, 89.7%; *p* = 0.009) and OAB group (MDI, 50.0%; LI, 41.7%; FUO, 81.8%; *p* = 0.005) but not in the W&W group (MDI, 71.4%; LI, 76.9%; FUO, 90.9%; *p* = 0.48). The 30-day mortality rate was highest in cases with MDI but the difference was not statistically significant (MDI, 14.3%; LI, 4.0%; FUO, 6.0%; *p* = 0.12). A total of 6 patients with MDI died. Of these, two had polymicrobial infections (1 case of *E. faecium* plus *P. aeruginosa* and 1 case of drug susceptible *P. aeruginosa* plus multidrug-resistant *P. aeruginosa*). The remaining 4 cases had infections with *S. epidermidis* (1 case), *E. faecium* (2 cases) and *S. aureus* (1 case). Cause of death was sepsis in all 6 cases. Patients with an ECOG PS of 2–4 had a significantly higher 30-day mortality rate than patients with an ECOG PS of 0–1 (42.9 vs. 5.2%; *p* = 0.007) although response rate did not differ significantly. Haematological/oncological diagnosis and remission status had no effect on the response rate, but patients with relapsed or refractory disease had a higher 30-day mortality rate than patients with newly diagnosed disease or in complete/partial remission. Median CRP level after failure of second-line treatment did not differ significantly between the three treatment groups (TGC, 127.0 mg/L; OAB, 135.4 mg/L; W&W, 150.0 mg/L; *p* = 0.18) and patients with a CRP level ≥ 100 mg/L did not have a significantly lower response compared to < 100 mg/L (66.9 vs. 77.6%; *p* = 0.14). However, in cases with a reduction in CRP level by > 60% (CrP ratio < 0.4), response rate was significantly higher (86.5 vs. 53.8%; *p* < 0.001) und 30-day mortality rate was significantly lower (1.0 vs. 10.8%; *p* = 0.01) than in cases with ≤ 60% reduction in CRP level.

**Table 4:** Response rate and 30-day mortality rate

Characteristic	Response *n*/*N* (%)	*p* value	Mortality *n*/*N* (%)	*p* value
Treatment group		0.12^a^		0.94^a^
TGC	67/91 (73.6)		7/91 (7.7)	
OAB	44/71 (62.0)		5/71 (7.0)	
W&W	30/38 (78.9)		2/38 (5.3)	
Sex		0.76^a^		0.42^a^
Male	74/107 (69.2)		9/107 (8.4)	
Female	67/93 (72.0)		5/93 (5.4)	
Age		0.005^a^		0.42^a^
< 60 years	66/107 (61.7)		5/93 (5.4)	
≥ 60 years	75/93 (80.6)		9/107 (8.4)	
ECOG PS^b^		0.09^a^		0.007^a^
0–1	128/173 (74.0)		9/173 (5.2)	
2–4	3/7 (42.9)		3/7 (42.9)	
Diagnosis		0.33^a^		0.06^a^
AML	83/122 (68.0)		4/122 (3.3)	
ALL	19/26 (73.1)		5/26 (19.2)	
HL	4/4 (100)		0/4	
GCT	1/2 (50.0)		0/2	
MDS/MPN	4/4 (100)		0/4	
MM	10/11 (90.9)		0/11	
NHL	20/31 (64.5)		5/31 (16.1)	
Remission status		0.76^a^		0.002^a^
ID	52/78 (66.7)		3/78 (3.8)	
CR	47/66 (71.2)		3/66 (4.5)	
PR	18/22 (81.8)		0/22	
Relapse	12/17 (70.6)		4/17 (23.5)	
Refractory disease	12/17 (70.6)		4/17 (23.5)	
Neutropenia^c^		0.09^a^		1.0^a^
Yes	114/168 (67.9)		12/168 (7.1)	
No	27/32 (84.4)		2/32 (6.3)	
Duration of neutropenia		0.35^a^		1.0^a^
< 21 days	76/103 (73.8)		7/103 (6.8)	
≥ 21 days	65/97 (67.0)		7/97 (7.2)	
Chemotherapy		0.13^a^		0.63^a^
Conventional CTx	94/140 (67.1)		11/140 (7.9)	
Autologous HSCT	23/32 (71.9)		1/31 (3.1)	
Allogeneic HSCT	24/28 (85.7)		2/28 (7.1)	
Cause of p/r fever		< 0.001^a^		0.12^a^
FUO	72/83 (86.7)		5/83 (6.0)	
MDI	25/42 (59.5)		6/42 (14.3)	
LI	44/75 (58.7)		3/75 (4.0)	
CRP level		0.14^a^		0.56^a^
< 100 mg/L	52/67 (77.6)		6/67 (8.9)	
≥ 100 mg/L	89/133 (66.9)		8/133 (6.0)	
CRP ratio		< 0.001^a^		0.01^a^
< 0.4	83/96 (86.5)		1/96 (1.0)	
≥ 0.4	50/93 (53.8)		10/93 (10.8)	

*AML* acute myeloid leukaemia, *ALL* acute lymphatic leukaemia, *CR* complete remission, *CRP* C-reactive Protein, *CTx* chemotherapy, *ECOG PS* Eastern Cooperative Oncology Group performance score, *FUO* fever of unknown origin, *GCT* germ cell tumour, *HL* Hodgkin lymphoma, *HSCT* haematopoietic stem cell transplantation, *ID* initial diagnosis, *LI* lung infiltrates, *MDI* microbiologically documented infection, *MDS* myelodysplastic syndrome, *MM* multiple myeloma, *MPN* myeloproliferative neoplasm, *NHL* non-Hodgkin lymphoma, *OAB* other-antibiotics, *PR* partial remission, *p/r* persistent or recurrent, *TGC* tigecycline, *W&W* watch & wait

^a^Pearson’s χ^2^ test

^b^in 11 cases (OAB group) and 9 cases (W&W group), no data was available regarding ECOG PS

^c^after failure of second-line treatment

### Resolution of neutropenia

Neutropenia had already resolved in 32 cases (16.0%) before p/r fever. In 93 cases (46.5%), defervescence occurred while patients were still neutropenic. Of these, neutropenia resolved within 7 days in 57 cases. Durable defervescence for 7 days was achieved in 36 cases (18.0%) even before resolution of neutropenia. Patients who were still neutropenic 7 days after failure of carbapenem had a worse response rate to third-line treatment than patients in which neutropenia had resolved (57.5 vs. 80.5%; *p* < 0.001). This was statistically significant for the TGC group (59.0 vs. 84.6%; *p* = 0.006) and OAB group (50.0 vs. 73.0%; *p* = 0.046), but not for the W&W group (71.4 vs. 83.3%; *p* = 0.39).

## Discussion

Although tigecycline is only approved for the treatment of intra-abdominal, complicated skin or soft-tissue infections, an implementation in the treatment of severe infections such as pneumonia, bacteraemia or febrile neutropenia has been observed. However, two meta-analyses, with 7434 and 7689 cases, respectively, found a worse outcome for tigecycline in the treatment of serious infections compared to the control group even for its approved indications [18, 19]. In bloodstream infections, tigecycline increased clinical response rates but mortality was not affected [11]. These studies mainly included immunocompetent patients. In one of the first retrospective studies regarding the use of tigecycline in immunocompromised patients, 110 cancer patients with serious infections received tigecycline, mostly as second line treatment. While 58% had a haematologic malignancy, only 28% were neutropenic. The response rate was 64% overall and 67% in neutropenic patients [12]. In a prospective observational study with 207 patients, 62.3% had a haematologic malignancy or solid tumour, but only 14% were neutropenic. The response rate for all neutropenic patients was not reported, but in 12 cases, empiric use of tigecycline in neutropenic patients showed a response rate of 58% for tigyecycline [20]. Mortality for neutropenic patients was not reported in both studies. A retrospective study with 35 patients focused on treatment of febrile neutropenia with tigecycline and showed a response rate of 43% and a mortality rate of 23% [13]. Interestingly, in our study, response rate to tigecycline was 73.6% and 30-day mortality rate was only 7.7%, although inclusion and response criteria were similar. This may be explained by the smaller number of patients in the study of Schwab et al. [13]. In addition, the amount of previous antibiotic treatments varied more (range 1–5) while in our study only cases with two previous treatments were included. In a recent retrospective study reporting on 73 patients with febrile neutropenia unresponsive to a carbapenem, tigecycline showed a fairly low response rate of 35% although the study design was comparable to ours. One reason could be, that in that study a response required a decrease in CRP in addition to defervescence. But similar to our study, no significant difference in response rate or 30-day mortality rate could be shown [21]. In one of the few prospective randomized controlled studies, tigecycline was combined with piperacillin/tazobactam as first-line treatment for febrile neutropenia and showed a higher response rate of 67.9% compared to piperacillin/tazobactam alone (44.3%). The mortality rate was similar in both groups. Interestingly 34 patients received tigecycline after failure of piperacillin/tazobactam monotherapy with a response rate of 69% in bacteraemia and 86% in FUO [22]. In another prospective study, 68% of 125 patients with febrile neutropenia responded to tigecycline as second-line therapy [14]. The response rates of most of these studies were similar and our study had comparable response rates to tigecycline of 73.6% overall and 69.4% in neutropenic patients.

While these results show that tigecycline is a reasonable treatment option for febrile neutropenia, most of the previous studies did not have a control group. In our study, we compared tigecycline to other antibiotics as third-line treatment after failure of a carbapenem (meropenem in almost all cases) or a watch & wait strategy. There was no significant difference in response rates or 30-day mortality rate between the three treatment groups. MDI was detected in 42 cases (21.0%), of which 38 cases (19.0%) were bloodstream infections. In a study of 687 febrile episodes, 245 episodes were considered breakthrough fever, in terms of p/r fever [6]. At 16.3%, the proportion of febrile episodes with MDI was similar in this study. Accordingly, a relevant proportion of patients develop MDI during antibiotic treatment. These results confirm the relevance of renewed microbiological diagnosis despite ongoing antibiotic therapy. A total of 44 bacterial pathogens were detected in this study. Of these, 75.0% were Gram-positive. The most common pathogens were CoNS and *Enterococcus* spp. In the Gram-negative spectrum, *E. coli* and *P. aeruginosa* were the most common. Although an increase in Gram-negative pathogens has been reported in febrile neutropenia in recent years [23, 24], the data mostly relates to the first episode of fever. However, in relapsed fever, the pathogen spectrum can be altered and fewer Gram-negative pathogens may be detectable compared to the first febrile episode [6]. It has to be noted, that in this study, third-line therapy was often started empirically. Looking the microbiological spectrum, it seems to be beneficial to wait for the results of the blood cultures and to adapt accordingly. In our study, the overall response rate of cases with MDI was 59.5% and significantly lower than with FUO (59.5 vs. 86.7%; *p* < 0.001) but comparable to cases with LI (58.7%). The TGC- and OAB group also showed a lower response rate in MDI and LI compared to FUO but not in the W&W group. Because of the few studies about third-line therapy and the small number of cases, a comparison regarding the response of the different causes of fever is difficult. However, other studies on febrile neutropenia also showed a better response rate for patients with FUO (50–92.9%) than with LI (25–61.3%) or with MDI (36.7–81.5%) [25–27]. Thirty-day mortality was highest in cases with MDI in this study, but the difference was not statistically significant (FUO, 6.0%; LI, 4.0%; MDI, 14.3%; *p* = 0.12). Mortality from bloodstream infections in cancer patients has been significantly reduced over the years (1978–2001) from approximately 25 to 6% [24], again looking mainly at first-line therapy studies. The results of this study may indicate that patients with p/r febrile neutropenia and MDI generally have a higher mortality rate. Nevertheless, because of the small number of cases (6/42), no definitive conclusion can be drawn. The high rate of defervescence in FUO in this study supports the recommendation of most guidelines not to switch antibiotic therapy if no cause of fever can be found [7]. Nevertheless, in p/r fever, thorough microbiologic and radiologic diagnostics should be performed in addition to clinical diagnostics to adequately diagnose and treat possible infections.

Because this study is retrospective it is unclear why some patients were not switched to third-line treatment, so some bias in patient selection cannot be ruled out, although the W&W group was equal in most characteristics compared to the other groups. Cause of p/r fever was not significantly different between the 3 groups but MDI tended to be more common in the W&W-group (TGC, 15.4%; OAB, 19.7%; W&W, 36.8%; *p* = 0.07), which is relevant because patients with MDI or LI had lower response rates than patients with FUO (MDI,. 59.5%; LI, 58.7%; FUO, 86.7%; *p* < 0.001). Several studies have investigated the early discontinuation of antibiotic treatment in neutropenic patients with FUO showing no increased mortality or admission to ICU [28, 29]. A switch to third-line treatment might therefore be unnecessary in these patients.

It should also be kept in mind that tigecycline must always be combined with at least one anti-pseudomonal antibiotic in the treatment of febrile neutropenia. In this study, tigecycline was combined with ceftazidime in 95.6% of cases therefore the therapeutic success cannot be attributed to one specific antibiotic. Also, tigecycline is mostly bacteriostatic which might be not sufficient in neutropenic patients. In summary, no advantage could be shown for the TGC group.

Both granulocytes and antibiotics are essential for treatment of febrile neutropenia [27]. On the other hand there is a well-known association between antibiotic consumption and the occurrence of multidrug-resistant bacteria [30, 31]. Therefore, efforts should be made to reduce antibiotic use in the management of febrile neutropenia [32]. A “watchful waiting” after failure of a second-line antibiotic treatment, as a result of our study, might be possible, especially in those patients who at the time of p/r febrile episodes were no longer neutropenic or who are expected to recover from neutropenia within few days. The recurrence of fever per se is not a strong predictor of severe infectious complications or death [33]. Therefore, in the sense of a resource-saving use of anti-infective drugs, the implementation of standard operating procedures should be demanded.

Based on this study, the undirected or “empiric” use of tigecycline plus an anti-pseudomonal antibiotic in p/r fever following chemotherapy-induced neutropenia and after failure of carbapenem therapy cannot generally be recommended. Especially in cases with neither LI nor MDI a watch and wait strategy may be warranted. However, this study has limitations due to its retrospective nature and the long time period during which changing resistance patterns and treatment options could affect the results. Further prospective, multicentre studies investigating the efficacy of tigecycline plus an anti-pseudomonal antibiotic in febrile neutropenia are needed. Until the benefit of a regular antibiotic switch in p/r fever is proven, reserve antibiotics, such as tigecycline, should be considered only selectively, to reduce the use of antibiotic and avoid resistance development.

## Supplementary Information

Below is the link to the electronic supplementary material.

Supplementary file1 (DOCX 54 KB)

Supplementary file2 (DOCX 19 KB)

## Data Availability

The data that support the findings of this study are available from the corresponding author upon reasonable request.

## Declarations

### Clinical trial registration

This retrospective chart review study was not registered within a clinical trial registry.
