# Supplementary material for: Tigecycline as salvage treatment of febrile neutropenia in patients with haematological malignancies—a retrospective single-centre analysis of 200 cases
Source: Ann Hematol. 2023 Apr 25;102(9):2607–16. doi: 10.1007/s00277-023-05222-5 (PMC10444688; doi:10.1007/s00277-023-05222-5)
Supplement: Supplementary file 1 — Supplementary file1 (DOCX 54 KB) [file 277_2023_5222_MOESM1_ESM.docx]

**
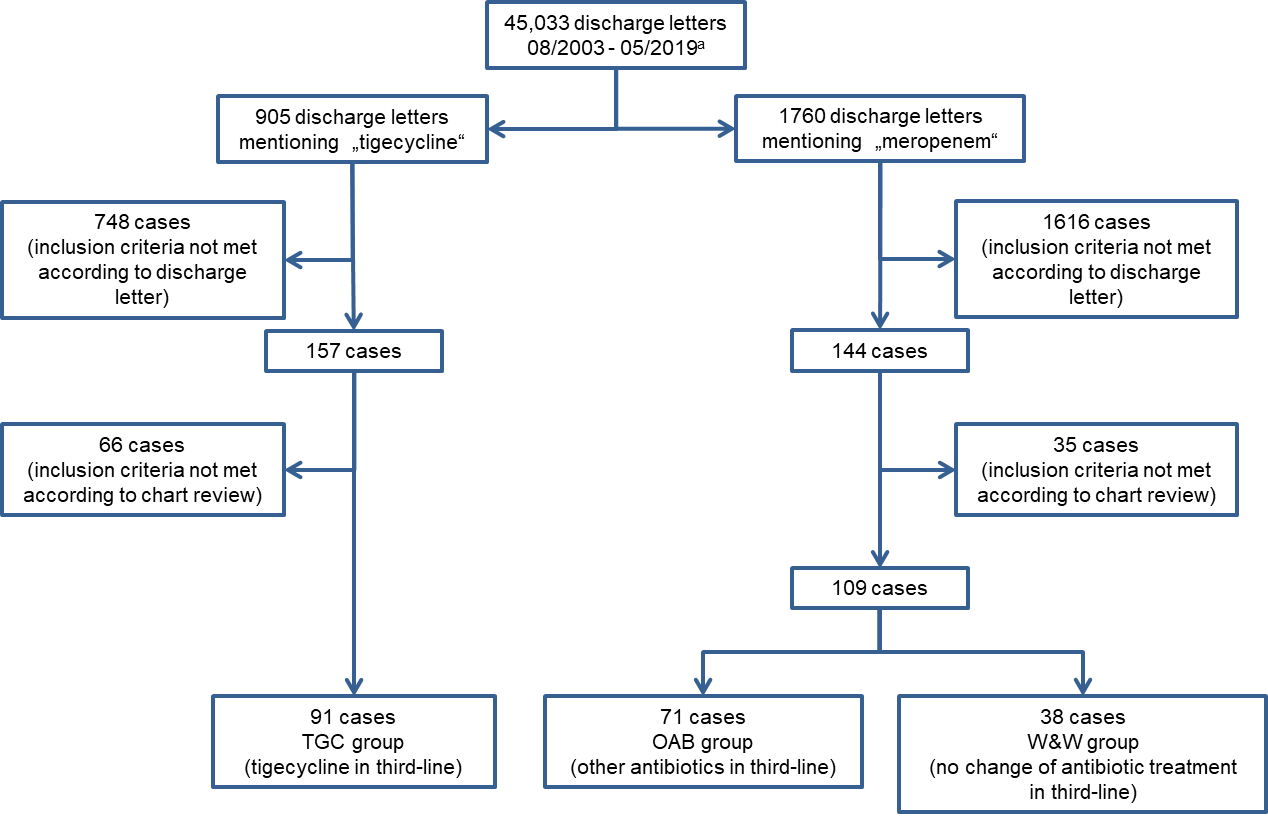
**

**Supplementary Figure 1** Study enrollment

*OAB* other-antibiotics, *TGC* tigecycline, *W&W* watch & wait

^a^cases identified that met inclusion criteria: only from 09/2004 to 04/2019
