# Supplementary material for: Tigecycline as salvage treatment of febrile neutropenia in patients with haematological malignancies—a retrospective single-centre analysis of 200 cases
Source: Ann Hematol. 2023 Apr 25;102(9):2607–16. doi: 10.1007/s00277-023-05222-5 (PMC10444688; doi:10.1007/s00277-023-05222-5)
Supplement: Supplementary file 2 — Supplementary file2 (DOCX 19 KB) [file 277_2023_5222_MOESM2_ESM.docx]

**Supplementary Table 1** Results of antibiotic susceptibility testing of positive blood cultures

| Antibiotic Sensitivity | TGC  *n*=13 | OAB  *n*=16 | W&W  *n*=14 |
| --- | --- | --- | --- |
| **Gram-positive, *n*** | **9** | **16** | **8** |
| Penicillin G | 0/6; 3 *nt* | 1/8; 8 *nt* | 2/5; 3 *nt* |
| Ampicillin | 0/5; 4 *nt* | 1/7; 8 *nt;*  1 *intermediate* | 1/2; 6 *nt* |
| Amoxicillin/clavulanic acid | 1/5; 4 *nt* | 1/11; 5 *nt* | 2/4; 4 *nt* |
| Vancomycin | 9/9 | 15/16 | 8/8 |
| Linezolid | 9/9 | 15/15; 1 *nt* | 7/7; 1 *nt* |
| Tigecycline | 7/7; 2 *nt* | 9/9; 7 *nt* | 4/4; 4 *nt* |
|  |  |  |  |
| **Gram-negative, *n*** | **4** | **0** | **6** |
| Piperacillin/tacobactam | 2/4 |  | 4/5;  1 *intermediate* |
| Meropenem | 1/3;  1 *intermediate* |  | 6/6 |
| Ciprofloxacin | 2/4 |  | 4/5;  1 *intermediate* |
| Ceftazidime | 3/4 |  | 4/5;  1 *intermediate* |
| Gentamicin | 2/4 |  | 6/6 |
| Tigecycline | 4 *nt* |  | 3/4; 2 *nt* |

*OAB* other-antibiotics, *TGC* tigecycline, *W&W* watch & wait, *nt* not tested
